# Seasonality of anti-*Leishmania infantum* titers in dogs: a crucial factor for designing effective clinical trials

**DOI:** 10.3389/fvets.2024.1477696

**Published:** 2024-10-01

**Authors:** Maria Alfonsa Cavalera, Oana Gusatoaia, Andrea Zatelli

**Affiliations:** Department of Veterinary Medicine, University of Bari, Bari, Italy

**Keywords:** canine, leishmaniosis, prevention, seroprevalence, study design, vector-borne disease

## 1 Introduction

Canine leishmaniosis (CanL), caused by *Leishmania infantum*, remains a significant focus in veterinary parasitology, with a worldwide distribution and an estimated 2.5 million infected dogs in southwestern Europe (1–4). From a public health perspective, this sandfly-borne disease also represents a significant global health issue due to its zoonotic nature (5).

Therefore, it is not surprising that research on CanL has grown exponentially over the past two decades, with over 3,900 scientific papers published on diverse aspects of the disease (PubMed database, accessed on 7 August 2024). This surge in scholarly activity underscores the complexity and importance of understanding CanL, from its epidemiology and pathophysiology to its treatment and prevention. Research efforts have particularly focused on the latter two areas, with several clinical trials being conducted to evaluate the efficacy of therapies and preventive measures for CanL. These efforts have been crucial for significantly reducing the disease burden and preventing the spread of the protozoan in endemic and non-endemic regions, respectively. To date, due to the various research endeavors, we know that the all-around control of *L. infantum* infection can be achieved through an integrating approach. This includes the use of sandfly repellents as well as three main areas of intervention: chemotherapy, immunotherapy, and immunoprophylaxis (6).

## 2 Subsection relevant to the subject

The World Association for the Advancement of Veterinary Parasitology (WAAVP) has always recognized the significance of leishmaniosis among canine vector-borne diseases (VBDs). In 2021, the WAAVP developed guidelines that provide comprehensive recommendations for conducting studies aimed at evaluating the efficacy of parasiticides in reducing vector-borne pathogen (VBP) transmission risks in dogs and cats (7). These guidelines serve as a valuable resource for researchers, pharmaceutical companies, and regulatory authorities involved in VBD research, including CanL (7). In this regard, according to the WAAVP guidelines, field studies aiming to assess the efficacy of products for preventing *L. infantum* transmission in companion animals should adhere to strict inclusion criteria (e.g., equal distribution between control and treated dogs, randomization, and allocation by household) (7). Moreover, animals should be followed up for at least 1 year, with assessments conducted before inclusion, at the end of the efficacy period of the investigational product, and at the end of the observational period (7). If feasible, intermediate assessments should be conducted every 3–4 months (7).

## 3 Discussion

Despite the thoroughness of the WAAVP guidelines, the present opinion article aims to focus on a crucial aspect of the host–parasite relationship that can have a significant impact on the design and results of clinical trials, particularly in regions with distinct climatic patterns: the seasonality of anti-*L. infantum* antibody titers in dogs. Indeed, shortly after the WAAVP guidelines were published, an article by Cavalera et al. showed that *L. infantum* antibody titers can vary significantly between the transmission and non-transmission seasons in dogs from a hyperendemic area for CanL (i.e., Apulia region, Southern Italy) (8). For the sake of clarity, it should be noted that in temperate regions, the transmission of *Leishmania* is highly seasonal, with higher infection rates during warmer months when sandflies are most active, the so-called “transmission period” or “sandfly season.” In the article cited above, most of the enrolled dogs (*n* = 36/65; 55.4%) experienced a reduction in anti-*L. infantum* antibody titers, as measured by the indirect fluorescent antibody test (IFAT), during the non-transmission season. Nearly half of these dogs (*n* = 16/36; 44%) became seronegative. Similarly, seasonal variations in *Leishmania* antibody titers during sand fly transmission and non-transmission periods were observed in domestic ferrets in Spain (9). It has been hypothesized that the reduction of anti-*L. infantum* antibody titers during the non-sand fly period may be related to the progressive reduction of exposure to vectors. More specifically, the immune response of the host could be upregulated during the transmission period because of uninfected and *L. infantum*-infected sand fly bites and the immunogenic effect of the parasite. It should be considered that the measurement of antibody titers in dogs is a crucial and ever-present practice in clinical/parasitological trials for the diagnosis and therapeutic monitoring of this parasitosis, as outlined in the currently available guidelines (10, 11). Moreover, among the serology techniques for *L. infantum*, IFAT remains the most suitable assay used for detecting anti-*L. infantum* antibodies, as recommended by the World Organization for Animal Health (12). Ignoring the seasonality of antibody titers can lead to significant biases in clinical trial results evaluating the efficacy of new therapeutic strategies or preventive measures as well as the prevalence/incidence of CanL in the canine population. For example, trials starting during the transmission season and ending during the non-transmission season could lead to an “inflated” efficacy of the molecule(s) under investigation, if any reduction in antibody titers is entirely (and wrongly) attributed to the treatment effect. Similarly, the assessment of the prevalence or incidence of CanL in a dog population may yield diametrically opposed results depending on the season chosen for the study.

With regard to the design of trials to evaluate products capable of preventing *L. infantum* infection, the authors believe that it would be advisable to perform the enrolment at the end of the non-transmission season and to conclude the study at the end of the next transmission season (considering a study period of 18 months). This approach would enable the inclusion of dogs that can be considered “truly *L. infantum* seronegative” at the outset of the study and allow for an assessment of how many of these dogs have actually been protected from exposure. An alternative approach would be to enroll clinically healthy *L. infantum* seropositive dogs (i.e., those previously exposed to the protozoan) at the end of the non-transmission season and evaluate them at the end of the following transmission season, taking advantage of the “seasonality effect.” If, at the end of the transmission season, anti-*L. infantum* antibody titers are elevated—even in the absence of clinical signs and laboratory abnormalities (such as increased C-reactive protein and/or ferritin, elevated total protein with hypergammaglobulinemia, and a decreased albumin/globulin ratio) consistent with CanL (11, 13–15)—it can be posited that the animal has been exposed to the sand fly bites despite the use of the repellent product.

In addition, it is important to consider that in both countries where the seasonal variation of *L. infantum* antibodies was detected (i.e., Italy and Spain), a confluent bi-modal trend in the seasonal dynamics of *Phlebotomus perniciosus* was observed (16). Therefore, before applying the suggested indications to set up clinical studies, it would be appropriate to consider the seasonal dynamics of the Mediterranean *L. infantum* vectors described (16).

In conclusion, seasonality of anti-*L. infantum* titers in dogs can represent a critical factor that should not be overlooked in the design of clinical trials aimed at evaluating treatments and preventive measures for CanL. Incorporating this variable will ensure more accurate and reliable results, which will ultimately contribute to more effective control strategies for this potentially life-threatening disease for dogs.
